# Vascular resistance index and the immediate hemodynamic success of lower limb distal artery revascularization

**DOI:** 10.1590/1677-5449.202301192

**Published:** 2024-03-04

**Authors:** Rebecca Paes de Andrade Souza Caldas, Esdras Marques Lins, Gabriela de Oliveira Buril, Fernanda Appolônio Rocha, Emmanuelle Tenório Albuquerque Godoi Berenguer de Barros e Silva, Larissa Barbosa de Andrade, Camilla Lins da Cunha Cavalcanti, Guilherme Barros Alves de Carvalho

**Affiliations:** 1 Universidade Federal de Pernambuco – UFPE, Hospital das Clínicas – HC, Empresa Brasileira de Serviços Hospitalares – EBSERH, Recife, PE, Brasil.; 2 Universidade Federal de Pernambuco – UFPE, Recife, PE, Brasil.; 3 Faculdade Pernambucana de Saúde – FPS, Recife, PE, Brasil.

**Keywords:** vascular resistance, Doppler ultrasonography, arteriography, peripheral arterial disease, ankle-brachial index

## Abstract

**Background:**

Revascularization surgery is used to attempt to restore blood flow to the foot in patients with critical ischemia (CI) caused by peripheral arterial occlusive disease of the lower limbs (LL). Ultrasonography with Doppler (USD) SAH emerged in recent years as a highly valuable method for planning this surgical intervention.

**Objectives:**

To evaluate the relationship between the resistance index (RI), measured with USD, and immediate hemodynamic success of LL revascularization surgery in patients with CI.

**Methods:**

The study design was a prospective cohort assessing 46 patients with LL CLI who underwent operations to perform infrainguinal revascularization by angioplasty or bypass from August 2019 to February 2022. All patients underwent preoperative clinical vascular assessment with USD including measurement of the RI of distal LL arteries, LL arteriography, and measurement of the ankle-brachial index (ABI). All patients had their ABI measured again in the immediate postoperative period.

**Results:**

Forty-six patients were assessed, 25 (54.3%) of whom were male. Age varied from 32 to 89 years (mean: 67.83). Hemodynamic success was assessed by comparison of preoperative and postoperative ABI, showing that hemodynamic success was achieved in 31 (67.4%) patients after revascularization surgery (ABI increased by 0.15 or more). A positive correlation (p ≤ 0.05) was observed between the RI of the distal revascularized LL artery and immediate hemodynamic success assessed by ABI (lower RI and hemodynamic success).

**Conclusions:**

This study observed a positive correlation between the resistance index of the distal artery and immediate hemodynamic success of lower limb revascularizations, as assessed by the ankle-brachial index, so that the lower the RI the greater the hemodynamic success achieved.

## INTRODUCTION

Critical ischemia (CI) of the lower limbs (LL) is the most severe stage of peripheral arterial occlusive disease (PAOD) and is related to high rates of morbidity and mortality. Although invasive, digital subtraction angiography (DSA) is the imaging exam most used to define management of patients who are candidates for LL revascularization surgery. Treatment planning using this examination should be based on a detailed analysis of the anatomy of the distal arterial circulation.^1-4^

In these cases, in addition to assessment with DSA, it is also possible to employ hemodynamic data for decision making. Ultrasonography with Doppler (USD) is a noninvasive tool that is available at the majority of services and can provide information on flow, velocity, and resistance in the distal arterial bed. However, these characteristics are often undervalued in preoperative assessment.^5,6^

Doppler ultrasound can be used to derive the resistance index (RI) of distal LL arteries. The RI is determined by subtracting the peak systolic velocity (PSV) from the end-diastolic velocity (EDV) and dividing by the PSV. As such, the greater the EDV, the lower the RI, inferring that the distal bed SAH lower resistance.^7^

Since the RI is a ratio, it is not affected by changes resulting from use of different angles to measure velocities with USD. This minimizes differences provoked when examinations are performed by different examiners. The RI is therefore an easily acquired tool that is of great value for studying peripheral resistance of the distal arterial bed.^7^

The status of the distal arterial bed SAH a direct impact on the results of revascularization surgery. However, the impact on the outcomes of this surgery of hemodynamic factors such as distal arterial resistance have been studied little. Arterial resistance can be measured transoperatively; but, in addition to being invasive and labor-intensive, these measurements are not available when the artery to be treated is being chosen. Doppler ultrasound is a useful and accessible option for evaluating the resistance of LL arteries noninvasively during the preoperative period.^8,9^

In view of these considerations, the objective of this study was to evaluate the relationship between the RI of distal arteries and the immediate hemodynamic success of infrainguinal revascularization surgery in patients with LL CI.

## METHODS

**Figure 1:**
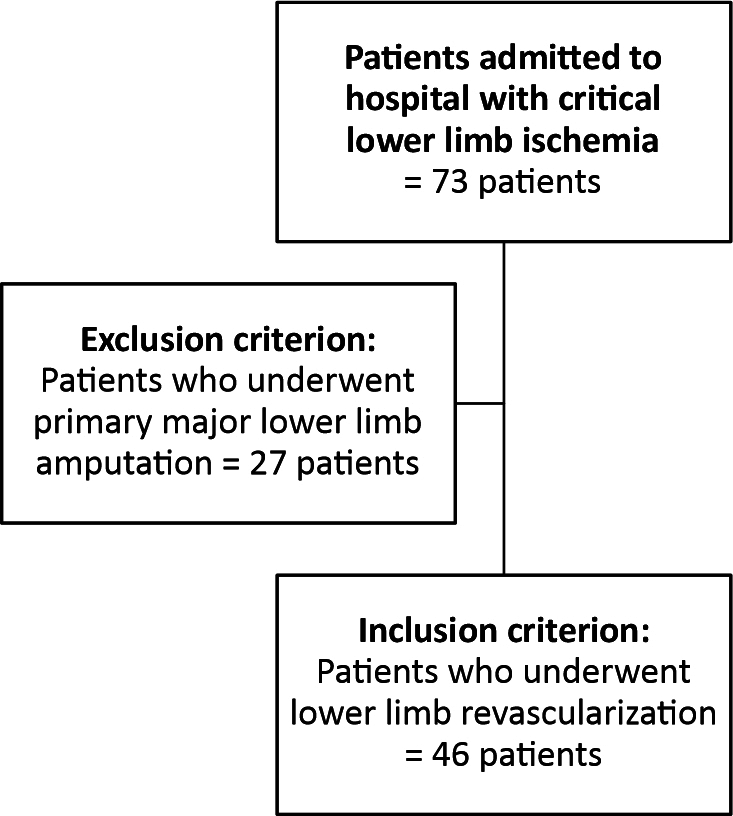
Patient flow diagram.

The study design was a prospective cohort. A total of 46 patients admitted for LL CI from August 2019 to February 2022 were assessed. All underwent clinical vascular assessment, were examined with USD and DSA, and had their ankle-brachial index (ABI) determined. Clinical vascular assessment included palpation of LL pulses and grading of degree of ischemia according to the Rutherford PAOD classification. Figure 1 presents a diagram illustrating the flow of patients through the study.

Doppler ultrasound was performed using GE brand equipment, models LOGIQ S7, LOGIQ P9, LOGIQ, and portable. Patients were examined in the supine position and RI values were derived for arterial segments distal of sites of stenosis or occlusion, providing flow was detected after optimizing the velocity, gain, and frequency parameters. The RI was calculated by dividing the difference between PSV and EDV by PSV (RI = PSV – EDV/PSV).

Each patient’s ABI was measured again after revascularization surgery was complete. The change in ABI was analyzed for each patient and those whose ABI had increased by 0.15 or more were defined as having had successful revascularization surgery from a hemodynamic point of view.^8^

Statistical analysis was performed using SPSS 13.0 for Windows and Excel 2019. All tests were executed with a 95% confidence interval. The RI variable was assessed for normality with the Kolmogorov-Smirnov test. The association between RI and surgical success/failure was analyzed using Student’s *t* test. The association between preoperative ABI and surgical success was analyzed with Fisher’s exact test. The sample size calculation employed the following Formula 1:


$$n={\left(\frac{\text{Z}\alpha /2\cdot \delta }{\text{E}}\right)}^{2}$$ (1)

where Zα/2 (the critical value for confidence) is 1.96; δ (standard deviation) is 0.13; and E (standard error) is 0.038 (5% of the mean, where the mean = 0.77).

This study was approved by the Human Research Ethics Committee at the Hospital das Clínicas da Universidade Federal de Pernambuco, affiliated to the Empresa Brasileira de Serviços Hospitalares (HC/EBSERH – UFPE), with Ethics Appraisal Submission Certificate 12346919.0.0000.8807. The study was written up in accordance with the STROBE guidelines.

## RESULTS

Twenty-five of the 46 patients assessed were male (54.3%). Age ranged from 32 to 89 years (mean of 67.83 and median of 69 years).

**Figure 2:**
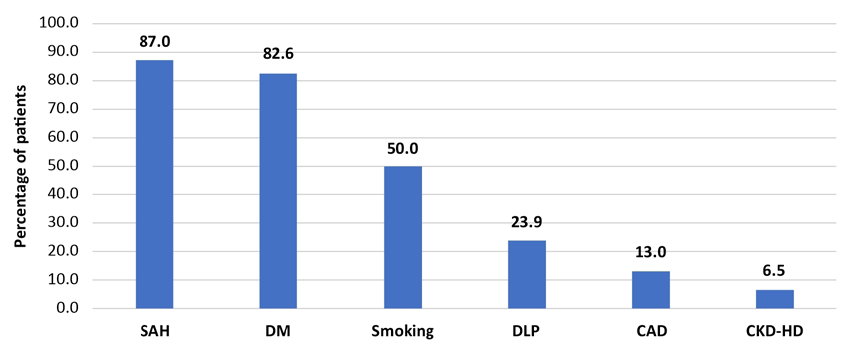
Comorbidities in the sample studied. SAH = systemic arterial hypertension; DM = diabetes mellitus; DLP = dyslipidemia; CAD = coronary artery disease; CKD-HD = chronic kidney disease on hemodialysis.

Prevalent risk factors for PAOD observed were systemic arterial hypertension (SAH), in 40 (87.0%) patients, diabetes mellitus (DM), in 38 (82.6%), and smoking (prior or current), in 23 (50.0%). All patients had obstructive atherosclerotic arteriopathy. There were no cases of obstructive arteriopathy secondary to inflammatory disease. Figure 2 illustrates the prevalence of comorbidities in the study patients.

With regard to the degree of lower limb ischemia, 41 (89.1%) patients were classified as Rutherford grade 5 (minor tissue loss). Four patients (8.7%) had major tissue loss, at Rutherford grade 6. One (2.2%) patient had pain at rest, at Rutherford grade 4.

Twenty (43.5%) of the patients assessed underwent open revascularization (bypass), 24 (52.2%) had their limbs revascularized by percutaneous transluminal angioplasty (PTA) and 2 (4.3%) underwent hybrid surgery (open surgery combined with endovascular techniques).

With regard to hemodynamic success, assessed by comparing preoperative and postoperative ABI, it was found that 31 (67.4%) patients had hemodynamically successful revascularization surgery (ABI increased by 0.15 or more) (Table 1).

**Table 1:** Correlation between preoperative ankle-brachial index (ABI) values and surgical success.

	Δ ABI (surgical success)		
Variables	Yes	No	p*
	n (%)	n (%)	
**Preoperative ABI**			
Incompressible (≥ 1.4)	0 (0.0)	8 (100.0)	**< 0.001**
Normal (0.8-1.4)	0 (0.0)	0 (0.0)	
Mild (0.6-0.79)	10 (66.7)	5 (33.3)	
Moderate (0.4-0.59)	12 (100.0)	0 (0.0)	
Severe (≤ 0.39)	9 (81.8)	2 (18.2)	

* Fisher’s exact test.

A positive correlation (p = 0.004) was observed between the RI of the distal revascularized lower limb artery and immediate hemodynamic success assessed by ABI (lower RI and hemodynamic success) (Table 2).

**Table 2:** Correlation between the resistance index (RI) and the hemodynamic success of revascularization surgery.

	Δ ABI (surgical success)		
Variables	Yes	No	Value de p*
	Mean ± SD	Mean ± SD	
RI of the distal artery	0.62**±**0.22	0.77**±**0.13	**0.004**

* Student’s *t* test.

ABI = ankle-brachial index; SD = standard deviation.

## DISCUSSION

This is the first study to relate the RI of the distal LL artery with the hemodynamic success of LL revascularization surgery. The RI measured using USD proved to be a measure that was easy to obtain and was reproducible, with a direct relationship with the hemodynamic success of the surgical procedure.

The search for means to improve the indications for revascularization surgery in patients with LL CI to achieve better results is a continuous need in vascular surgery. Peripheral resistance is a factor that influences the hemodynamic result achieved and should be considered during surgical planning. This study attempted to assess in conjunction the impact of assessing peripheral resistance with USD on the hemodynamic result of surgery.^10^

It is important to point out that the majority of people with LL CI are severe cases, with other associated comorbidities, such as DM, SAH, dyslipidemia, smoking, coronary artery disease, and chronic renal failure. Preoperative cardiological assessment habitually classifies these patients as at high risk of perioperative cardiac complications. Against this background, more optimized indications for surgical procedures, based on preoperative assessment tools, would translate to a higher success rate and, therefore, lower reintervention rates, reducing exposure of the patient to a new risk of cardiovascular events provoked by secondary surgery.^11^

Although all of the patients studied had LL CI, just 1/4 of the sample had ABI ≤ 0.39, which is compatible with severe ischemia. On the other hand, almost all of the study subjects had Rutherford grade 5 or 6. Only one case had pain at rest, which is still a severe stage of ischemia. As such, it is observed that not all of the patients exhibited ABI compatible with their severity. This was probably because of a considerable number of patients with falsely high ABI due to incompressibility, albeit partial, of the distal LL arteries. Patients with DM, particularly type 2, have thickening of the tunica media of the arteries. In some cases, this thickening leads to incapacity to compress the vessel because of calcium deposited in the area.^12,13^

In 2016, Tanno et al. evaluated use of a new measure, the ankle hemodynamic index, which demonstrated better correlation with the degree of ischemia compared to the ABI. This finding shows that hemodynamic information, such as peripheral arterial resistance, should be evaluated in conjunction with the ABI to provide a better preoperative analysis.^14^

Measurement of change in ABI was used to evaluate the hemodynamic success of revascularization surgery. In 2015, Je et al.^15^ found that revascularized patients with ABI change ≥ 0.15 exhibited, over the short term, improved symptoms and better daily functional capacity, including longer walking distance.

In turn, Katsuki et al.^8^ and Almasri et al.^16^ evaluated the long term outcomes and observed that ABI change ≥ 0.15 was an independent factor related to lower risk of additional revascularizations, limb loss, and mortality. In the present study, immediate hemodynamic success was observed in almost 70% of the study population. This value is within the average for data on patency (1 year after revascularization surgery) described in the literature.

It was observed that patients with lower RI subjected to revascularization of arteries tend to have better immediate hemodynamic results, according to change in ABI. Several studies that have assessed peripheral arterial resistance invasively during the intraoperative period show that lower resistance is related to better surgical success. However, few studies have evaluated ultrasonographic measures of this resistance with the objective of assessing revascularization outcomes.^17,18^

The value of USD for assessment of the distal LL recipient arteries for by-pass or angioplasty is well-established in the current literature, but, in the majority of cases, only velocity, flow, or anatomic data are considered. Assessment using the RI is simple and easy to reproduce across different examiners, since, as it is a ratio of velocities, there are no significant changes when different Doppler angles are used.^19^

Objective and noninvasive determination of resistance to identify which routes lead to better perfusion of the foot is important to predict the bets postoperative results. This was demonstrated by Buril,^20^ in 2022, who compared RI with images of the distal LL arteries obtained using DSA.

The major limitation of this study was the lack of follow-up of the patients for a longer period, which would have made it possible to establish the relationship between RI and the long term results of revascularization surgery, considering that the true success of limb revascularization cannot be measured merely by the ABI and must be monitored in terms of clinical progress, including improvement in pain and perfusion, presence of pulses and healing of ulcers. Therefore, additional studies, with larger sample and longer follow-up should be performed to confirm the benefit of systematic assessment of this index during planning of surgical LL revascularizations.

Another limitation of the study was that it was restricted to a single center. This was done to avoid errors in measuring RI, considering that this method is not yet widespread.

## CONCLUSIONS

In the present study, a positive correlation was observed between the RI of the distal artery and the immediate hemodynamic success of LL revascularization surgery, assessed by measurement of the ABI. As such, the lower the RI, the greater the hemodynamic success achieved.

This study presents a new way of assessing the distal lower limb arteries of patients with critical ischemia. Measurement of the resistance index as an adjuvant method can provide information of importance to the choice of which artery should be revascularized. Later studies, which are already ongoing, will be able to provide further data on the role of this index in determination of the success of lower limb revascularization surgery.
