# Supplementary material for: Vascular resistance index and the immediate hemodynamic success of lower limb distal artery revascularization
Source: J Vasc Bras. 2024 Mar 4;23:e20230119. doi: 10.1590/1677-5449.202301192 (PMC10939178; doi:10.1590/1677-5449.202301192)
Supplement: Translation

# Translation: Relação entre o índice de resistência vascular e o sucesso hemodinâmico de revascularizações distais dos membros inferiores

**DOI:** 10.1590/1677-5449.202301191

**Authors:** Rebecca Paes de Andrade Souza Caldas, Esdras Marques Lins, Gabriela de Oliveira Buril, Fernanda Appolônio Rocha, Emmanuelle Tenório Albuquerque Godoi Berenguer de Barros e Silva, Larissa Barbosa de Andrade, Camilla Lins da Cunha Cavalcanti, Guilherme Barros Alves de Carvalho

**Affiliations:** 1 Universidade Federal de Pernambuco – UFPE, Hospital das Clínicas – HC, Empresa Brasileira de Serviços Hospitalares – EBSERH, Recife, PE, Brasil.; 2 Universidade Federal de Pernambuco – UFPE, Recife, PE, Brasil.; 3 Faculdade Pernambucana de Saúde – FPS, Recife, PE, Brasil.

**Keywords:** resistência vascular, ultrassonografia Doppler, arteriografia, doença arterial periférica, índice tornozelo-braquial

## Abstract

**Contexto:**

A cirurgia de revascularização é proposta para restaurar o fluxo sanguíneo para o pé nos casos de isquemia crítica (IC) devido a doença arterial obstrutiva periférica dos membros inferiores (MMII). O uso de ultrassonografia com Doppler (USD) vem despontando nos últimos anos como um método de grande valor para o planejamento cirúrgico dessa intervenção.

**Objetivos:**

Avaliar a relação entre o índice de resistência (IR), mensurado por meio de USD, e o sucesso hemodinâmico imediato da cirurgia de revascularização dos MMII em pacientes com IC.

**Métodos:**

O tipo de estudo empregado foi a coorte prospectiva, na qual foram avaliados 46 pacientes portadores de IC dos MMII submetidos à operação de revascularização infrainguinal por angioplastia ou em ponte de agosto de 2019 a fevereiro de 2022. Todos os pacientes foram submetidos à avaliação clínica vascular, à USD com medida do IR das artérias distais dos MMII, à arteriografia dos MMII e à aferição do índice tornozelo-braquial (ITB) no período pré-operatório. No pós-operatório imediato, todos os pacientes foram submetidos à nova aferição do ITB.

**Resultados:**

Entre os 46 pacientes avaliados, 25 (54,3%) eram do sexo masculino. A idade variou de 32 a 89 anos (média de 67,83). Quanto ao sucesso hemodinâmico, avaliado pela comparação do ITB pré e pós-operatório, constatou-se que 31 (67,4%) pacientes apresentaram sucesso hemodinâmico após cirurgia de revascularização (aumento do ITB em 0,15 ou mais). Foi observada correlação positiva (p ≤ 0,05) entre o IR da artéria distal revascularizada do MMII e o sucesso hemodinâmico imediato avaliado pela aferição do ITB (IR menor e sucesso hemodinâmico).

**Conclusões:**

Na presente pesquisa foi observada uma correlação positiva entre o índice de resistência arterial distal e o sucesso hemodinâmico nas revascularizações dos membros inferiores, avaliada através do índice tornozelobraquial, de forma que, quanto menor foi o IR, maior o sucesso hemodinâmico obtido.

## INTRODUÇÃO

A isquemia crítica (IC) dos membros inferiores (MMII) é o estágio mais grave da doença arterial obstrutiva periférica (DAOP) e está relacionada à morbidade e mortalidade altas. A arteriografia por subtração digital (ASD), embora invasiva, é o exame de imagem mais utilizado na definição da conduta em pacientes candidatos à cirurgia de revascularização do MMII. O planejamento terapêutico com esse exame deve ser baseado em uma análise detalhada da anatomia da circulação arterial distal^1-4^ .

Neste cenário, além da avaliação com a ASD, é possível também utilizar dados hemodinâmicos para a tomada de decisão. A ultrassonografia com Doppler (USD) é uma ferramenta não invasiva e que está disponível na maioria dos serviços, podendo fornecer informações de fluxo, velocidade e resistência do leito arterial distal. Porém, tais características são frequentemente subvalorizadas na avaliação pré-operatória^5,6^ .

Por meio da USD, pode ser obtido o índice de resistência (IR) das artérias distais dos MMII. Ele é determinado pelo resultado da subtração da velocidade de pico sistólico (VPS) da velocidade diastólica final (VDF) dividida pela VPS. Dessa forma, quanto maior a VDF, menor é o índice, inferindo menor resistência do leito distal^7^ .

Por se tratar de uma razão de valores, o IR não sofre as alterações provocadas pela utilização de diferentes ângulos na obtenção das velocidades na USD. Desta forma, minimizam-se as diferenças obtidas quando os exames são realizados por diferentes examinadores. Assim, o IR torna-se uma ferramenta de fácil obtenção e de grande valor para o estudo da resistência periférica do leito arterial distal^7^ .

A situação do leito arterial distal influencia diretamente nos resultados da cirurgia de revascularização. No entanto, os impactos de fatores hemodinâmicos no desfecho desta cirurgia, como a resistência arterial distal, ainda são pouco estudados. As medidas de resistência arterial podem ser obtidas no transoperatório; porém, além de invasivas e laboriosas, não são aferidas antes da escolha da artéria a ser abordada. A USD é uma alternativa útil e acessível para avaliar a resistência em artérias dos MMII de forma não invasiva no pré-operatório^8,9^ .

A partir dessas considerações, o objetivo deste estudo foi avaliar a relação entre o IR das artérias distais e o sucesso hemodinâmico imediato de cirurgias de revascularização infrainguinal em pacientes com IC dos MMII.

## MÉTODOS

O modelo de estudo empregado foi a coorte prospectiva. Foram avaliados 46 pacientes internados com IC dos MMII no período de agosto de 2019 a fevereiro de 2022. Todos foram submetidos à avaliação clínica vascular, à USD, à ASD e à aferição do índice tornozelo-braquial (ITB). A avaliação clínica vascular envolveu a palpação de pulsos dos MMII e a caracterização do grau de isquemia através da classificação de Rutherford para DAOP. A Figura 1 mostra o fluxograma dos pacientes do estudo.

**Figura 1:** Fluxograma de pacientes.

A USD foi realizada em equipamentos da marca GE, nos modelos LOGIQ S7, LOGIQ P9 e LOGIQ E portátil. Os pacientes foram avaliados em decúbito dorsal, e foram obtidos os valores de IR nas artérias em segmentos distais aos locais de estenose ou oclusão, desde que houvesse fluxo identificado após otimizados os parâmetros de velocidade, ganho e frequência. O IR foi calculado através da fórmula obtida pela divisão da diferença da VPS e da VDF pela VPS (IR = VPS – VDF/VPS).

Após a realização de cirurgia de revascularização, o ITB foi novamente aferido. A variação do ITB foi analisada em cada indivíduo, e aqueles que obtiveram um aumento igual ou maior que 0,15 foram considerados como tendo apresentado sucesso da cirurgia de revascularização do ponto de vista hemodinâmico^8^ .

Para análise estatística, foram utilizados os *softwares* SPSS 13.0 para Windows e o Excel 2019. Todos os testes foram aplicados com intervalo de confiança de 95%. A variável IR foi avaliada pelo teste de normalidade de Kolmogorov-Smirnov. A associação do IR com o sucesso ou insucesso cirúrgico foi realizada através do teste *t* de Student. A associação entre o ITB pré-operatório e o sucesso cirúrgico foi realizada através do teste exato de Fisher. O cálculo do tamanho da amostra foi feito utilizando a seguinte Fórmula 1:


$$n={\left(\frac{\text{Z}\alpha /2\cdot \delta }{\text{E}}\right)}^{2}$$ (1)

em que Zα/2 (o valor crítico para o grau de confiança) é 1,96; δ (desvio-padrão) é 0,13; e E (erro-padrão) é 0,038 (5% da média, sendo a média = 0,77).

Este estudo foi aprovado pelo Comitê de Ética em Pesquisa em Seres Humanos (CEP) do Hospital das Clínicas da Universidade Federal de Pernambuco vinculado à Empresa Brasileira de Serviços Hospitalares (HC/EBSERH – UFPE), sob o registro do Certificado de Apresentação de Apreciação Ética 12346919.0.0000.8807. A redação do artigo seguiu a diretriz STROBE.

## RESULTADOS

Entre os 46 pacientes avaliados, 25 eram do sexo masculino (54,3%). A idade variou de 32 a 89 anos (média de 67,83 anos e mediana de 69).

Em relação aos fatores de risco para DAOP, foram observados como prevalentes a hipertensão arterial sistêmica (HAS), presente em 40 (87,0%) pacientes, o diabetes melito (DM), em 38 (82,6%), e o tabagismo (pregresso ou ativo), em 23 (50,0%). Todos os pacientes apresentavam arteriopatia obstrutiva aterosclerótica. Não houve casos de arteriopatia obstrutiva por doença inflamatória. Na Figura 2, é apresentada a prevalência das comorbidades no grupo de pacientes do estudo.

**Figura 2:** Comorbidades na amostra estudada. HAS = hipertensão arterial sistêmica; DM = diabetes melito; DLP = dislipidemia; DAC = doença arterial coronariana; DRC-HD = doença renal crônica em hemodiálise.

Quanto ao grau de isquemia do membro inferior, 41 (89,1%) pacientes foram classificados como tendo grau 5 de Rutherford (lesão tecidual menor). Quatro pacientes (8,7%) apresentavam lesão tecidual maior, com grau 6 de Rutherford. Um (2,2%) paciente apresentava dor em repouso, com grau 4 de Rutherford.

Entre os pacientes avaliados, 20 (43,5%) foram submetidos à revascularização aberta (ponte), 24 (52,2%) tiveram o membro revascularizado através de angioplastia transluminal percutânea (ATP) e 2 (4,3%) foram submetidos a cirurgia híbrida (técnica aberta associada à endovascular).

Quanto ao sucesso hemodinâmico, avaliado através da comparação do ITB pré e pós-operatório, foi constatado que 31 (67,4%) pacientes apresentaram sucesso hemodinâmico da cirurgia de revascularização (aumento do ITB em 0,15 ou mais) (Tabela 1).

**Tabela 1:** Correlação entre valores do índice tornozelo-braquial (ITB) no pré-operatório e o sucesso cirúrgico.

	Δ ITB (sucesso cirúrgico)		
Variáveis	Sim	Não	Valor de p^*^
	n (%)	n (%)	
**ITB pré-operatório**			
Incompressível (≥ 1,4)	0 (0,0)	8 (100,0)	**< 0,001**
Normal (0,8-1,4)	0 (0,0)	0 (0,0)	
Leve (0,6-0,79)	10 (66,7)	5 (33,3)	
Moderado (0,4-0,59)	12 (100,0)	0 (0,0)	
Grave (≤ 0,39)	9 (81,8)	2 (18,2)	

* Teste exato de Fisher.

Foi observada uma correlação positiva (p = 0,004) entre o IR da artéria distal revascularizada do membro inferior e o sucesso hemodinâmico imediato avaliado pela aferição do ITB (IR menor e sucesso hemodinâmico) (Tabela 2).

**Tabela 2:** Correlação entre o índice de resistência (IR) e o sucesso hemodinâmico da cirurgia de revascularização.

	Δ ITB (sucesso cirúrgico)		
Variáveis	Sim	Não	Valor de p^*^
	Média ± DP	Média ± DP	
IR da artéria distal	0,62**±**0,22	0,77**±**0,13	**0,004**

* Teste *t* de Student.

ITB = índice tornozelo-braquial; DP = desvio-padrão.

## DISCUSSÃO

Este foi o primeiro estudo a relacionar o IR da artéria distal dos MMII com o sucesso hemodinâmico de uma cirurgia de revascularização dos MMII. O IR aferido através da USD apresentou-se como uma medida de fácil obtenção e reprodutibilidade, tendo relação direta com o sucesso hemodinâmico do procedimento cirúrgico.

Buscar formas de aperfeiçoar a indicação de cirurgias de revascularização em pacientes com IC dos MMII para obter melhores resultados é uma necessidade contínua na área de cirurgia vascular. A resistência periférica é um fator que influencia no resultado hemodinâmico obtido e deve ser considerada durante o planejamento cirúrgico. Este estudo buscou avaliar de forma conjunta o impacto da avaliação da resistência periférica através da USD no resultado hemodinâmico dessas cirurgias^10^ .

É importante salientar que os pacientes portadores de IC dos MMII são, em sua maioria, pacientes graves, com outras comorbidades associadas, como DM, HAS, dislipidemia, tabagismo, doença arterial coronariana e insuficiência renal crônica. A avaliação cardiológica pré-operatória habitualmente classifica esses pacientes como tendo alto risco para complicações cardíacas perioperatórias. Diante desse contexto, a indicação mais otimizada de procedimentos operatórios, por meio do uso de ferramentas de avaliação no pré-operatório, implicaria em um maior índice de sucesso e, portanto, em menores taxas de reintervenção, reduzindo a exposição do paciente a novo risco de eventos cardiovasculares derivados de cirurgias secundárias^11^ .

Apesar de todos os pacientes estudados serem portadores de IC dos MMII, apenas 1/4 da amostra apresentou ITB ≤ 0,39, sendo compatível com isquemia grave. Por outro lado, quase todos os indivíduos estudados avaliados apresentaram classificação de Rutherford 5 ou 6. Apenas um caso apresentou dor em repouso, sendo também considerado um estágio grave de isquemia. Assim, observa-se que nem todos os pacientes apresentaram ITB compatível com a gravidade. Isso provavelmente está relacionado a um número relevante de pacientes com ITB falsamente elevado, devido a incompressibilidade, ainda que parcial, das artérias distais dos MMII. Os pacientes portadores de DM, sobretudo tipo 2, apresentam espessamento da camada média das artérias. Em alguns casos, esse espessamento acarreta a incapacidade de comprimir o vaso devido ao depósito de cálcio na região^12,13^ .

Tanno et al.^14^ , em 2016, avaliaram a utilização de uma nova medida, o índice hemodinâmico do tornozelo, que, comparado ao ITB, demonstrou melhor correlação com o grau de isquemia. Esse achado demonstra que informações hemodinâmicas, como resistência arterial periférica, devem ser avaliadas junto com o ITB para uma melhor análise pré-operatória.

Através da mensuração da variação do ITB, foi avaliado o sucesso hemodinâmico imediato da cirurgia de revascularização. Em 2015, Je et al.^15^ identificaram que pacientes revascularizados com variação de ITB ≥ 0,15 apresentaram, em curto prazo, melhora dos sintomas e maior capacidade funcional em seu dia a dia, incluindo maior distância caminhada.

Katsuki et al.^8^ e Almasri et al.^16^ , por sua vez, avaliaram o desfecho a longo prazo e observaram que a variação de ITB ≥ 0,15 é um fator independente relacionado a menor risco de novas revascularizações, perda do membro e mortalidade. No presente estudo, o sucesso hemodinâmico imediato foi observado em quase 70% da população estudada. O valor encontrado está dentro da média dos dados de perviedade (após 1 ano da cirurgia de revascularização) descritos na literatura.

Foi observado que pacientes submetidos à revascularização de artérias com IR mais baixo tendem a apresentar melhor resultado hemodinâmico imediato, de acordo com a variação do ITB. Diversos estudos que avaliaram a resistência arterial periférica de forma invasiva no intraoperatório mostraram que uma menor resistência está relacionada a um melhor sucesso cirúrgico. Porém, poucos trabalhos avaliam medidas ultrassonográficas dessa resistência com a finalidade de avaliar o desfecho da revascularização^17,18^ .

O valor da USD para avaliação das artérias distais dos MMII receptoras de um *by-pass* ou de uma angioplastia é bem estabelecido na literatura atual, mas, na maioria das vezes, são considerados apenas os valores de velocidade, fluxo ou dados anatômicos. A avaliação realizada através do IR é simples e de fácil reprodutibilidade entre examinadores distintos, uma vez que, por tratar-se de um índice de velocidades, não há alterações significativas quando utilizados ângulos diferentes no Doppler^19^ .

A determinação objetiva e não invasiva da resistência para identificar quais caminhos levam a uma melhor perfusão do pé é importante para predizer um melhor resultado no pós-operatório. Isso foi demonstrado por Buril^20^ , em 2022, ao compararem o IR com as imagens das artérias distais dos MMII obtidas através da ASD.

A principal limitação da atual pesquisa foi a falta de acompanhamento dos pacientes por um período maior, possibilitando estabelecer a relação entre o IR e o resultado da cirurgia de revascularização a longo prazo, considerando que o real êxito de uma revascularização de membro não pode ser aferida apenas com uma medida do ITB, devendo ser acompanhada pela evolução clínica, como melhora da dor e da perfusão, presença de pulsos e cicatrização das lesões. Por isso, mais estudos, com maior amostra e seguimento durante maior tempo, podem ser realizados para que se reforce o benefício da avaliação sistemática desse índice no planejamento cirúrgico das revascularizações de MMII.

Outra limitação da pesquisa foi a sua realização em único centro médico. Isso ocorreu para evitar erros na aferição do IR, considerando que o uso desse método ainda não está estabelecido.

## CONCLUSÃO

No presente estudo, foi observada correlação positiva entre o IR da artéria distal e o sucesso hemodinâmico imediato da cirurgia de revascularização do MMII, avaliado pela aferição do ITB. Dessa forma, quanto menor foi o IR, maior foi o sucesso hemodinâmico obtido.

Esta pesquisa apresenta uma nova forma de avaliar as artérias distais dos MMII. A aferição do índice de resistência como método coadjuvante pode fornecer informações importantes na escolha da artéria que será revascularizada. Estudos posteriores, já em andamento, poderão fornecer mais dados sobre o papel desse índice na determinação do sucesso da cirurgia de revascularização dos membros inferiores em pacientes com isquemia crítica.
